# Knowledge of CT exposure parameters among Norwegian student radiographers

**DOI:** 10.1186/s12909-020-02233-y

**Published:** 2020-09-14

**Authors:** Sundaran Kada

**Affiliations:** Faculty of Health and Social Sciences, Western Norway University of Applied Sciences, Post Box 7030, 5020 Bergen, Norway

**Keywords:** CT exposure parameters, Optimization, Radiation dose, Image quality, Student radiographers, Survey

## Abstract

**Background:**

Improvements in the competency levels of student radiographers in computed tomography examinations (CT) are important due to the increasing number of these examinations being undertaken in imaging departments. The present study assesses the knowledge of student radiographers regarding CT exposure parameters.

**Methods:**

The level of knowledge related to CT exposure parameters was evaluated using a twenty-one-item questionnaire that was distributed to final-year student radiographers. The questionnaire consisted of questions around CT exposure parameters and either allowed respondents to answer “true,” or “false” or choose a response from a range of responses where only one answer was correct. Correct answers were given one mark, while no mark was given for an incorrect answer. The score out of possible 21 was converted to a percentage, with a higher percentage signifying greater knowledge.

**Results:**

Seventy-two students completed and returned the questionnaire, resulting in a 71% response rate. The mean score was 53%. Only 33% of students correctly identified that kilovoltage peak (kVp) should be increased when patients have metallic implants, and milliampere seconds (mAs) should be increased as body part thickness increases. No one answered all the questions correctly. There was no significant knowledge difference between students who had CT facilities on campus and those that did not.

**Conclusion:**

Overall, student radiographers’ knowledge of CT exposure parameters was reported to be satisfactory.

## Background

Developments in the technical aspects of computed tomography (CT) technology, such as multidetector CT, iterative reconstruction algorithms, and dual-energy CT, have resulted in increased scan speed and improved image quality [1, 2]. This progress has resulted in a twofold increase in the use of CT examinations in the clinical environment over the past three decades [3]. CT is the most commonly used imaging modality in Norway [4], and its use doubled (11–21%) from 2002 to 2008 [5]. CT examinations account for about 20% of all radiological examinations in Norway and has the highest use of CT procedures among Nordic countries [6]. CT scans remain popular due to their capabilities [7], despite contributing significantly to ionizing radiation exposure for medical use. In fact, CT is the greatest source of exposure for the United States population, contributing to almost one-half of total radiation exposure for medical use [8, 9] and accounting for almost 60% of the radiation exposure from imaging modalities in western countries [10]. In Norway, CT examinations contributed to 66% of the total radiation dose received due to medical examinations in 2002 rising to 79% in 2008 [5]. It is estimated three new cases of cancer occur annually in Norway due to CT examinations [11]. Therefore the ‘as low as reasonably achievable’ (ALARA) principle must be practiced to ensure that the benefits from undertaking the scan are always greater than the possible risks [12].

There are six radiography schools in Norway. Radiography education in Norway involves a three-year degree program and one-third of this time is spent in clinical practice. Teaching of radiography students is undertaken by lecturers in the academic settings and by supervising clinical radiographers in the clinical settings. On average 20 h of lectures are provided on CT related topics in the university setting, including 2–4 h of lectures specifically related to exposure parameters. In addition, all students must have a minimum of 5 weeks CT clinical placement in radiology departments during their study program. Theoretical competency is assessed via written examination during the first and second year, and clinical competence is assessed by a clinical supervising radiographer at the end of the students’ clinical placement period. Only one of the universities that provides radiography education in Norway has CT machine in its skills laboratories (skills labs) on campus.

CT exposure parameters, such as kilovoltage peak (kVp), milliampere seconds (mAs), pitch, slice thickness, automatic tube current modulation (ATCM), detector configuration, and reconstruction algorithms, control radiation dose and image quality [13]. There are several possible combinations of these parameters available that enables the radiographer to select a protocol that provides optimal image quality along with a reduced radiation dose. Some combinations can be manufacture specific, however, the standard settings and manufacturer recommendations may be designed for an average sized patient aimed at optimizing image quality rather than radiation dose [14]. CT dose reduction requires a combination of strategies including, optimization of scanning protocols, justification of CT use, decrease of unnecessary examinations, and better training and education for radiographers [15]. Dose optimization contributes to significant reduction in radiation dose to adults [15] and understanding CT exposure parameters is a prerequisite for dose optimization [16]. For example, any change in kVp affects the image noise, contrast and patient dose [17], mAs determines image noise and patient dose [18], ATCM reduces radiation dose by up to 25–50% in children and adults [19, 20], and iterative reconstruction algorithms is reported to be a new era in CT optimization providing high quality images with 28–98% dose reduction [21]. Therefore, there is a need to understand the appropriate strategies to optimize CT examinations in order to comply with the ALARA principle. Once examinations are justified, radiographers select the best combination of imaging parameters for each individual patient to achieve sufficient image quality to provide a diagnosis with the lowest radiation dose to the patient. It is therefore necessary to have sound knowledge about CT exposure parameters and their effects on both image quality and radiation dose to develop an optimization protocol. A lack of understanding will therefore limit the potential for optimization [22]. Studies have described radiographers’ self-reported competency on CT exposure parameters [23–25]. However, to the author’s knowledge, no studies have been conducted nationally or internationally to assess student radiographers’ level of knowledge of exposure parameters in CT. Accordingly, the present study assesses the knowledge of student radiographers’ regarding CT exposure parameters. In addition, the study explores if there is an association between students’ level of knowledge and the availability of a CT machine in the skills labs on campus. It is hypothesized that students generally have a good knowledge of CT exposure parameters and students with access to a CT machine on campus will have a higher level of knowledge than students who do not.

## Methods

### Design and subjects

This was a cross-sectional questionnaire-based study that was performed in Norway, which has a population of nearly 7 million people. Subjects were student radiographers in their final semester of the study program, prior to graduating in June 2019. Three of the five schools that have no CT on campus were randomly selected to participate in this study. All students from the one school that has CT equipment on campus were also included in this study. Therefore, students from four schools of radiography were invited to participate.

### Procedure

Heads of the schools of radiography that were to be included in this study, were contacted to request their consent to participate. After approval, questionnaires with an information letter were sent to the study coordinators to distribute them, collect them at the end of a teaching session, and send them back to author. Students were reminded to complete the questionnaires based on their own knowledge and not to use books or other sources when completing the questionnaire. The information letter explained the aim of the project, providing a guarantee of confidentiality (with regard to personal information), emphasizing the voluntary nature of participation, and finally, notifying the participants that they could access the results of the study by contacting the author. Data was collected in March 2019. Verbal consent was taken.

### Sample

A total of 72 final-year student radiographers from a possible sample of 101 participated (27 students from a possible sample of 32 from schools that have CT equipment and 45 students from a possible sample of 69 from schools that do not have CT equipment). At the time the study was conducted all students had undertaken their clinical placement in CT (a minimum of 5 weeks).

### Measures

Knowledge related to CT exposure parameters was measured using the Norwegian translation (translated, translated back and retranslated) of the English version of the questionnaire. The English version of the questionnaire was developed specifically to measure knowledge levels on CT exposure parameters and published in earlier studies [23, 24]. However, these questionnaires were not validated. The Norwegian translation version of the questionnaire was piloted to ten subjects. Based on suitability and feedback from the pilot study, the Norwegian version of the questionnaire contains two sections (Appendix). The first section consists of demographic information (whether the university has its own CT machine on campus, the number of weeks undertaking CT clinical placement, and confidence in their knowledge about changing CT exposure parameters in relation to radiation dosage and image quality). The second section consists of twenty-one questions related to CT exposure parameters (kVp, mAs, ATCM, pitch, slice thickness). The answers were measured using a two-point scale: “true/ false” or in a multiple-choice format where there is only one correct answer. Correct answers were given one mark, while no mark was given for an incorrect answer. Unanswered questions were scored as incorrect. The total score ranges are from 0 to 21, which were converted to a percentage. The Norwegian association of higher education institutions description of valuation criteria [26] was used to explain the students’ level of knowledge (Table 1). Students’ scores were compared with this valuation criteria to assess level of knowledge.

**Table 1:** General description of valuation criteria

Symbol	Description	General, qualitative description of valuation criteria
A	Excellent	An excellent performance, clearly outstanding. The candidate demonstrates excellent judgement and a very high degree of independent thinking
B	Very good	A very good performance. The candidate demonstrates sound judgement and a high degree of independent thinking
C	Good	A good performance in most areas. The candidate demonstrates a reasonable degree of judgement and independent thinking in the most important areas
D	Satisfactory	A satisfactory performance, but with significant shortcomings. The candidate demonstrates a limited degree of judgement and independent thinking
E	Sufficient	A performance that meets the minimum criteria, but no more. The candidate demonstrates a very limited degree of judgement and independent thinking
F	Fail	A performance that does not meet the minimum academic criteria. The candidate demonstrates an absence of both judgement and independent thinking

Symbol A represents mark between 88 and 100%, symbol B represents mark between 76 and 87%, Symbol C represents mark between 64 and 75%, symbol D represents mark between 52 and 63%, symbol E represents mark between 40 and 51% and symbol F represents mark below 40%

### Ethical considerations

The study followed the standard ethical guidelines for research conducted on students in Norway. Students were informed that participation was voluntary, and they were guaranteed anonymity and confidentiality. Approvals from the Medical Research Ethical Committee and the Norwegian Social Science Data Services were not required for this study.

### Statistical analysis

Descriptive statistics (frequency, percentage) are presented for demographic characteristics and mean, confidence interval (CI) for the summary score. Independent t-tests were conducted to compare the knowledge scores between students with or without confidence in their knowledge of CT exposure parameters and between students with and without CT equipment on campus. The relationship between knowledge scores and the students’ confidence in their knowledge was assessed using the Pearson correlation coefficient test. A *p*-value of less than 0.05 was considered to be statistically significant. Analyses were performed using the Statistical Package for the Social Sciences (SPSS), version 25.0.

## Results

A total of 101 questionnaires were distributed, and 72 students returned the questionnaire, resulting in a 71% response rate. Demographic characteristics of student radiographers including whether the school has its own CT machine on campus, the number of weeks in CT clinical placement, and confidence with knowledge in changing CT parameters in relation to radiation dosage and image quality are shown in Table 2.

**Table 2:** Demographic characteristics of participants (*n* = 72)

Variable	N (%)
CT on campus	
Yes	27 (38)
No	45 (62)
CT clinical placement	
1–5 weeks	26 (36)
6–10 weeks	42 (59)
11–15 weeks	3 (4)
16–20 weeks	1 (1)
Confidence with knowledge	
Very confident	3 (5)
Moderately confident	32 (44)
Less confident	32 (44)
Not confident	5 (7)

The summary scores for the students’ knowledge was 53%. Twenty-six students (36%) answered more than 50% incorrect. Only 24 (33%) correctly answered that kVp should be increased for patients with metallic implants and mAs should be increased as body part thickness increases. No one answered all the questions correctly. The students’ responses to each of the questions are presented in Table 3.

**Table 3:** Frequency distributions of responses to questionnaires (n = 72)

Questions	True (%)	False (%)
1. Reducing kVp would reduce the contrast resolution	41 (57)	31(43)
2. Increasing kVp by 50% is equivalent to doubling the mAs	39 (54)	33 (46)
3. kVp should be increased with patients having metallic implants	24 (33)	48 (67)
4. Doubling the mAs doubles the dose	55 (76)	17 (24)
5. Reducing the mAs reduces the noise	52 (72)	20 (28)
6. mAs should be increased as the body part thickness increases	24 (33)	48 (67)
7. ATCM is affected by improper patient positioning	51 (71)	21 (29)
8. ATCM decreases patient dose	49 (68)	23 (32)
9. ATCM increases the dose to obese patients	39 (54)	33 (46)
10. Decreasing the pitch degrades image quality	29 (40)	43 (60)
11. Increasing the pitch decreases the dose	38 (53)	34 (47)
12. Increasing the slice thickness decreases the dose	37 (51)	35 (49)
13. Decreasing the slice thickness reduces partial volume artefact	39 (54)	33 (46)
14. Increasing mAs decreases noise	57 (79)	15 (21)
15. Increasing kVp decreases noise	36 (50)	36 (50)
16. Increasing slice thickness increases noise	34 (47)	38 (53)
17. Increasing pitch increases noise	28 (39)	44(61)
18. A smoothing reconstruction kernel, increases the visualization of noise	25 (35)	47 (65)
19. Wider window settings, reduce the image contrast but also the visual perception of noise	27 (37)	45 (63)
20. Increasing the kVp from 120 to 140 kVp causes an increase in CTDI values of:	50 (69)	22 (31)
21. Which is the most informative index regarding the amount of dose that the patient would receive by the end of the examination??	28 (39)	44 (61)

No significant differences in knowledge scores were identified between students who reported being very/moderately confident (mean score 57%) and less/not confident in their knowledge (mean score 49%, *p* = 0.053) and between students from the school with CT equipment and schools without such equipment on campus (mean score 55% versus mean score 52%, *p* = 0.541) (Table 4). The Pearson correlation between students’ levels of confidence in their knowledge and the CT exposure parameter knowledge score was 0.073 (*p* = 0.051).

**Table 4:** CT exposure parameter knowledge scores

Characteristic	Mean score (95% CI)	P-value
Total group	53% (41–65%)	
Study place		0.541
CT on campus	55% (43–67%)	
No CT on campus	52% (40–64%)	
Confidence of knowledge		0.053
Very/moderately confident	57% (45–69%)	
Less/not confident	49% (37–61%)	

## Discussion

Overall, the current study indicates a satisfactory level of knowledge about CT exposure parameters (as measured by the mean score of 53%). According to the Norwegian Association of higher education institutions valuation criteria, marks between 52 and 63% indicates that performance is satisfactory, but with significant shortcomings; candidates demonstrate a limited degree of judgement and independent thinking [26]. The study findings are discouraging and raise concerns about knowledge levels related to CT exposure parameters among student radiographers prior to graduation. The title “radiographer” reflects the education and knowledge required to work safely in the field of diagnostic radiology. A possible explanation for the present findings could be that admission to radiography programs in Norway do not require high levels of knowledge in science subjects such as physics, mathematics, or chemistry. In the author’s experience, most students who lack this prerequisite knowledge find it difficult to cope with science-related subjects such as radiation physics and CT. Another possible explanation could be that supervising radiographers have not received specific training for their role. It has been indicated that training in supervision is a requirement for supervising staff [27]. Supervision is defined as, ‘the provision of guidance and feedback on matters of personal, professional and educational development in the context of a trainee’s experience of providing safe and appropriate patient care’ [28]. The supervising staff need to be clinically competent, knowledgeable and have good teaching and interpersonal skills to impart their knowledge effectively [29]. Furthermore, a student radiographer’s clinical practice takes place in a frequently busy, unpredictable context. Clinical staff are often distracted with their daily work schedule and may not have the time to allocate to teaching, which in turn hinders a student’s clinical competency [30]. A strong support is therefore required to enable effective and safe progression of a student’s learning in the clinical setting [31].

The finding that there was no significant difference in knowledge scores between those students that have CT equipment on campus and those that do not is dramatic and surprising. One might expect that the students from the school with a CT machine on campus would be more exposed to practical assignments where students develop protocols with various combinations of parameters, which would help solidify their theoretical and clinical knowledge. This study finding is inconsistent with earlier studies that identified more knowledge when skills labs are available on campus among medical students [32–34] and student radiographers [35]. Skills labs help students to bridge the gap between knowledge and practice and makes theoretical teaching “come alive” [36]. They also ensure that students acquire the necessary techniques and are properly assessed before practicing on real patient [37]. There are certain skills that cannot be practiced in real life-situations due to ethical reason. For example, training on real patients is not possible due to the harmful ionizing radiation involved. Clinical skills labs solve this problem by providing a safe environment for students to learn, practice and be observed performing skills in a simulated environment, thereby alleviating the risks involved in direct patient exposure [38]. There is no explanation for the present study finding. However, a potential reason could be that little time is allocated in skills labs for practical assignments or there is a lack of feedback from academic staff. In an internal evaluation, students reported a lack of feedback from academic staff for assignments. It has been demonstrated that feedback enhances trainees to reflect upon their actions, realize errors and to individually evaluate their learning progress [39]. As the subjects are future registered radiographers, they should be taught about CT exposure parameters so that they can effectively determine the examination protocol to be used during CT procedures to avoid excessive radiation dose to patients.

The study findings identify a lack of theoretical knowledge around the effects of CT exposure parameters on image quality and radiation dose. Therefore, the content of this theoretical study should be reviewed and updated. It is also recommended that radiography schools in Norway provide additional physics and math’s classes prior to CT related lectures and more case-based learning methods to improve students’ performance in this area of practice. Combined practical and theoretical knowledge leads to better outcomes in the teaching field and empowers more self-confidence of those working in the profession. Such a combined approach to teaching would enable learners to work more competently and be prepared to take responsibility in their future careers [40]. Knowledgeable and well-trained students play an important role in the creation of a positive radiation safety culture. Medical imaging, which is becoming increasingly complex and has an important impact on diagnosis [41], is an excellent example of a field in which competence is required [42–44].

## Strengths and limitations

Four out of six (67%) radiography schools in Norway participated in the current study, with a response rate of 71%. Therefore, this study concludes that the sample group is representative, which means that the results can be generalized as being indicative of the general knowledge of final-year student radiographers in Norway. A possible limitation of this study is that the questionnaires were distributed by study coordinators, which might have affected the degree to which participants were informed about the study, even though information letters were sent along with the questionnaires. Furthermore, there is no confirmation that the missing questionnaires were distributed or whether student radiographers were present during the time of distribution.

## Conclusion

The present study has shown that student radiographers have a satisfactory knowledge that demonstrates a very limited degree of judgement and independent thinking. Additionally, the student radiographers from the school with a CT machine on campus are not as knowledgeable about CT exposure parameters as might be expected.

### Implications for practices

The educational curriculum needs to be revised and updated with the importance of knowledge about CT exposure parameters emphasized during lectures. Radiographers that supervise students should be provided with specific training on the supervision of students and their role in this. Supervising radiographers should increase the amount of attention they give to these areas by providing specific knowledge around CT exposure parameters during mentorship and supervision. All radiography schools must strive to have skills labs with CT machines and more experimental assignments on phantoms should be conducted, to increase understanding of exposure parameters. It is essential that students are educated and supported in the development of their skills and knowledge in this area because of the increasing number of CT examinations globally and the risks associated with CT examinations.

Further studies are required to determine the benefits of having a CT machine on campus and to understand why students do not think these opportunities help them develop judgement and decision-making skills.

## Supplementary information

**Additional file 1.**

## Data Availability

The datasets used and/or analyzed during the current study are available from the corresponding author on reasonable request.

## Abbreviations

CT: Computed tomography
ALARA: As low as reasonably achievable
kVp: Kilovoltage peak
mAs: Milliampere seconds
ATCM: Automatic tube current modulation
SPSS: Statistical package for the social sciences
CI: Confidence interval
