# Supplementary material for: Knowledge of CT exposure parameters among Norwegian student radiographers
Source: BMC Med Educ. 2020 Sep 14;20:302. doi: 10.1186/s12909-020-02233-y (PMC7491127; doi:10.1186/s12909-020-02233-y)
Supplement: Supplementary file 1 — Additional file 1. [file 12909_2020_2233_MOESM1_ESM.docx]

**Appendix; Questionnaire**

**Section 1**

1. Does your study place have a CT machine in the skills labs on school campus?
   Yes 𖠹 No𖠹
2. Number of weeks you had in CT clinical placement during your study period:

1-5 weeks 𖠹 6-10 weeks 𖠹 11- 15 weeks 𖠹 16-20 weeks𖠹

1. How would you rate your confidence to alter the CT parameters correctly, considering image quality and radiation dose?

   Very confident 𖠹 Moderately confident 𖠹 Less confident 𖠹

Not confident 𖠹

**Section 2.**

**kVp**

|  |  | True | False |
| --- | --- | --- | --- |
| 4 | Reducing kVp would reduce the contrast resolution |  |  |
| 5 | Increasing kVp by 50% is equivalent to doubling the mAs |  |  |
| 6 | kVp should be increased with patients having metallic implants |  |  |

**mAs**

|  |  | True | False |
| --- | --- | --- | --- |
| 7 | Doubling the mAs doubles the dose |  |  |
| 8 | Reducing the mAs reduces the noise |  |  |
| 9 | mAs should be increased as the body part thickness increases |  |  |

**ATCM**

|  |  | True | False |
| --- | --- | --- | --- |
| 10 | ATCM is affected by improper patient positioning |  |  |
| 11 | ATCM decreases patient dose |  |  |
| 12 | ATCM increases the dose to obese patients |  |  |

**Pitch**

|  |  | True | False |
| --- | --- | --- | --- |
| 13 | Decreasing the pitch degrades image quality |  |  |
| 14 | Increasing the pitch decreases the dose |  |  |

**Slice thickness**

|  |  | True | False |
| --- | --- | --- | --- |
| 15 | Increasing the slice thickness decreases the dose |  |  |
| 16 | Decreasing the slice thickness reduces ‘partial volume’ artefacts |  |  |

**Noise**

|  |  | True | False |
| --- | --- | --- | --- |
| 17 | Increasing the mAs decreases noise |  |  |
| 18 | Increasing kVp decreases noise |  |  |
| 19 | Increasing slice thickness increases noise |  |  |
| 20 | Increasing pitch increases noise |  |  |
| 21 | A smoothing reconstruction kernal, increases the visualization of noise |  |  |
| 22 | Wider window settings, reduce the image contrast but also the visual perception of noise |  |  |

23. Increasing the kVp from 120-140 kVp causes an increase in CTDI values of:

17% 𖠹 38% 𖠹 65% 𖠹 89% 𖠹

24. Which is the most informative index regarding the amount of dose that the patient would receive by the end of the examination?

Computed Tomography dose index (CTDI) 𖠹
 Dose-length product (DLP) 𖠹
 Effective dose 𖠹
